# The effect of liver enzymes on adiposity: a Mendelian randomization study

**DOI:** 10.1038/s41598-019-52489-8

**Published:** 2019-11-14

**Authors:** Junxi Liu, Shiu Lun Au Yeung, Man Ki Kwok, June Yue Yan Leung, Shi Lin Lin, Lai Ling Hui, Gabriel Matthew Leung, C. Mary Schooling

**Affiliations:** 1 School of Public Health, Li Ka Shing Faculty of Medicine, The University of Hong Kong, Hong Kong SAR, China; 2 Department of Paediatrics, Faculty of Medicine, the Chinese University of Hong Kong, Hong Kong SAR, China; 3 City University of New York Graduate School of Public Health and Health Policy, New York, NY USA

**Keywords:** Type 2 diabetes, Risk factors

## Abstract

Poorer liver function is positively associated with diabetes in Mendelian randomization (MR) studies. Observationally, adiposity is associated with poorer liver function. To clarify the etiology, we assessed the association of liver enzymes with adiposity observationally and using two-sample MR for validation. In the “Children of 1997” birth cohort, we used multivariable linear regression to assess the associations of alanine transaminase (ALT) and alkaline phosphatase (ALP) at ~17.5 years with body mass index (BMI) (n = 3,458). Using MR, genetic predictors of ALT, ALP and gamma glutamyltransferase (GGT), were applied to genome-wide association studies of BMI (n = 681,275), waist circumference (WC) (n = 224,459) and waist-hip ratio (WHR) (n = 224,459) to obtain unconfounded estimates. Observationally, ALT was positively associated with BMI (0.10 kg/m^2^ per IU/L, 95% confidence interval (CI) 0.09 to 0.11). ALP was inversely associated with BMI (−0.018 kg/m^2^ per IU/L, 95% CI −0.024 to −0.012). Using MR, ALT was inversely associated with BMI (−0.14 standard deviation per 100% change in concentration, 95% CI −0.20 to −0.07), but not WC or WHR. ALP and GGT were unrelated to adiposity. Poorer liver function might not cause adiposity; instead higher ALT might reduce BMI, raising the question as to the role of ALT in body composition.

## Introduction

Observationally, poorer liver function, particularly nonalcoholic fatty liver disease (NAFLD), is associated with higher risk of type 2 diabetes mellitus (T2DM)^1^, but these studies are difficult to interpret because of the difficulty of distinguishing between correlated measures of liver function and the possibility of confounding by poor health causing both poor liver function and T2DM^2^. Recently, Mendelian randomization (MR) studies, taking advantage of the random allocation of genetic endowment at conception to obtain un-confounded estimates^3^, have clarified the role of liver function in T2DM. Specifically, these studies suggest that higher alanine aminotransferase (ALT)^4,5^ or aspartate aminotransferase (AST)^5^ rather than other measures of liver function, such as glutamyltransferase (GGT)^4–6^, could play a role in T2DM, although one small MR study found no association of ALT with T2DM^7^. Adiposity is also a very well-established cause of T2DM^8,9^. Whether specifically poor hepatocyte function relates to adiposity and contributes to T2DM, and by what mechanism is not entirely clear, although within an evolutionary biology framework we have previously suggested a mechanism via sex hormones^4^. Circulating levels of endogenous sex hormones are associated with both adiposity^10^ and fatty liver^11^. Observationally, poor liver function is associated with obesity^12^, but these studies are open to confounding by lifestyle, including diet and physical activity, health status, and socioeconomic position (SEP). As such, whether poor liver function is an additional contributor to the obesity epidemic remains uncertain, as experimental evidence is lacking.

To inform this important public health question as to whether liver function plays a role in obesity, we conducted two complimentary analyses with different assumptions and study designs. Observationally, we examined the association of liver function indicated by ALT and alkaline phosphatase (ALP) with adiposity in young people in a setting (Hong Kong) with little clear socio-economic patterning of obesity, so as to reduce confounding by poor health and SEP using Hong Kong’s “Children of 1997” birth cohort^13^. We also used an MR study to assess the effects of genetically predicted liver enzymes (ALT, ALP, and GGT)^14^ on adiposity indices, i.e., body mass index (BMI), waist circumference (WC), and waist-hip ratio (WHR), using the Genetic Investigation of ANthropometric Traits (GIANT) consortium^15–17^, overall and by sex.

## Results

### Children of 1997

**Figure 1:**
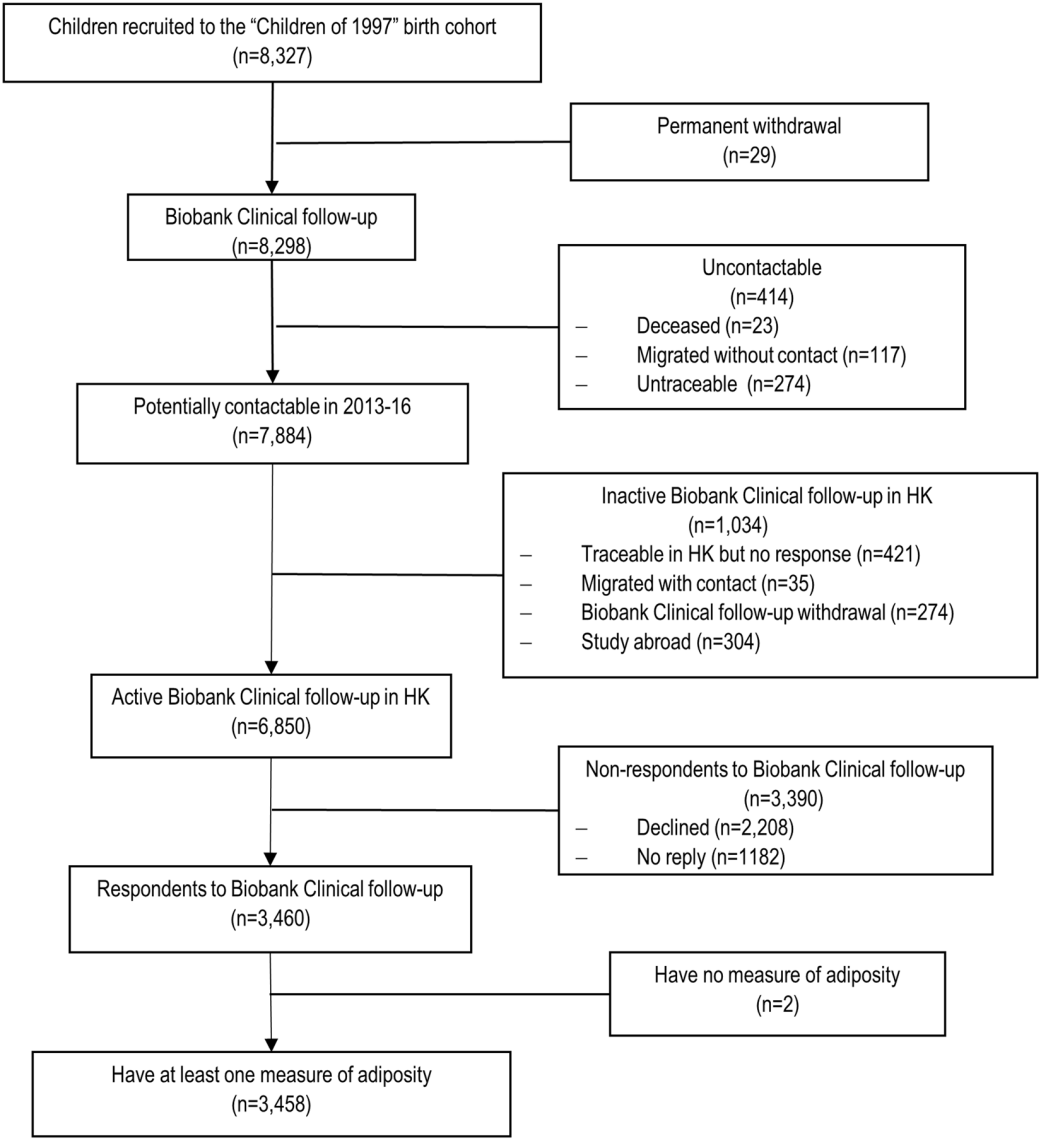
Flow chart of Hong Kong’s “Children of 1997” birth cohort Biobank Clinical follow-up, Hong Kong, China, 1997 to 2016.

In the “Children of 1997” Biobank Clinical follow-up, 3,460 of 6,850 potentially active follow-up participants took part (51% follow-up). 3,458 had at least one measure of BMI, WC, or WHR, as shown in Fig. 1. The 4,869 participants without adiposity measures were not different from the included participants in terms of sex, second-hand and maternal smoking exposure, and SEP with relatively small Cohen effect sizes (<0.13) (Supplemental Table S1). The mean and standard deviation (SD) of BMI, WC, and WHR were 20.9 kg/m^2^ (SD 3.5 kg/m^2^), 72.3 cm (SD 9.2 cm), and 0.77 (SD 0.06). Boys had higher BMI, WC, and WHR than girls. Maternal smoking was associated with larger BMI and WC. SEP had little association with BMI, WC, or WHR (Table 1).

**Table 1:** Baseline characteristics by body mass index (BMI), waist circumference (WC), and waist-hip ratio (WHR) among participants in Hong Kong’s “Children of 1997” birth cohort, Hong Kong, China, 1997 to 2016.

Characteristics	BMI (kg/m^2^)				WC (cm)				WHR			
	No.	%	Mean (SD)	P-value^*^	No.	%	Mean (SD)	P-value^*^	No.	%	Mean (SD)	P-value^*^
BMI (kg/m^2^)	3457		20.9 (3.5)	—		—		—	—	—	—	—
WC (cm)	—	—	—	—	3453	—	72.3 (9.2)	—	—	—	—	—
WHR	—	—	—	—	—	—	—	—	3452	—	0.77 (0.06)	—
Sex	3457	—	—	<0.001	3453	—	—	<0.001	3452	—	—	<0.001
Girl	1716	49.6%	20.7 (3.3)	—	1713	49.6%	69.7 (7.8)	—	1712	49.6%	0.75 (0.05)	—
Boy	1741	50.4%	21.2 (3.8)	—	1740	50.4%	74.9 (9.7)	—	1740	50.4%	0.79 (0.07)	—
Unknown	0	—	—	—	0	—	—	—	0	—	—	—
Second-hand and maternal smoking exposure	3457	—	—	<0.001	3453	—	—	<0.01	3452	—	—	0.05
None	943	27.3%	20.6 (3.1)	—	943	27.3%	71.6 (8.3)	—	942	27.3%	0.77 (0.06)	—
Prenatal second-hand smoking	1280	37.0%	20.9 (3.7)	—	1278	37.0%	72.3 (9.5)	—	1278	37.0%	0.77 (0.06)	—
Postnatal second-hand smoking	961	27.8%	21.3 (3.8)	—	959	27.8%	73.0 (9.8)	—	959	27.8%	0.77 (0.07)	—
Maternal smoking	128	3.7%	21.5 (3.4)	—	128	3.7%	73.6 (9.4)	—	128	3.7%	0.78 (0.06)	—
Unknown	145	4.2%	20.6 (3.1)	—	145	4.2%	71.0 (7.8)	—	145	4.2%	0.76 (0.06)	—
Highest parental education level	3457	—	—	0.71	3453	—	—	0.39	3452	—	—	0.29
Grade < =9	991	28.7%	21.0 (3.8)	—	990	28.7%	72.6 (9.8)	—	990	28.7%	0.77 (0.07)	—
Grades 10–11	1489	43.1%	20.9 (3.4)	—	1486	43.0%	72.2 (8.8)	—	1485	43.0%	0.77 (0.06)	—
Grades > =12	961	27.8%	20.9 (3.5)	—	961	27.8%	72.3 (9.3)	—	961	27.8%	0.77 (0.06)	—
Unknown	16	0.5%	20.8 (2.0)	—	16	0.5%	69.2 (5.1)	—	16	0.5%	0.74 (0.04)	—
Highest parental occupation	3457	—	—	0.01	3453	—	—	0.07	3452	—	—	0.42
I (unskilled)	99	2.9%	20.7 (3.1)	—	99	2.9%	71.6 (8.9)	—	99	2.9%	0.77 (0.06)	—
II (semiskilled)	285	8.2%	21.3 (3.7)	—	284	8.2%	72.8 (9.3)	—	284	8.2%	0.77 (0.07)	—
III (semiskilled)	503	14.6%	20.8 (3.5)	—	503	14.6%	72.2 (9.4)	—	503	14.6%	0.77 (0.07)	—
III (nonmanual skilled)	879	25.4%	21.0 (3.7)	—	878	25.4%	72.5 (9.4)	—	877	25.4%	0.77 (0.06)	—
IV (managerial)	440	12.7%	21.4 (3.8)	—	440	12.7%	73.3 (10.2)	—	440	12.7%	0.77 (0.07)	—
V (professional)	797	23.1%	20.7 (3.3)	—	797	23.1%	71.8 (8.7)	—	797	23.1%	0.77 (0.06)	—
Unknown	454	13.1%	20.7 (3.4)	—	452	13.1%	71.6 (8.3)	—	452	13.1%	0.77 (0.06)	—
Household income per head at recruitment	3457	—	—	0.12	3453	—	—	0.14	3452	—	—	0.45
First quintile	572	16.5%	20.8 (3.5)	—	571	16.5%	71.9 (9.0)	—	571	16.5%	0.77 (0.07)	—
Second quintile	616	17.8%	21.0 (3.7)	—	614	17.8%	72.4 (9.9)	—	614	17.8%	0.77 (0.07)	—
Third quintile	618	17.9%	21.2 (3.7)	—	617	17.9%	73.2 (9.6)	—	617	17.9%	0.77 (0.06)	—
Fourth quintile	631	18.3%	20.8 (3.4)	—	631	18.3%	72.1 (8.8)	—	631	18.3%	0.77 (0.06)	—
Fifth quintile	646	18.7%	20.8 (3.2)	—	646	18.7%	72.0 (8.6)	—	645	18.7%	0.77 (0.07)	—
Unknown	374	10.8%	21.1 (3.7)	—	374	10.8%	72.2 (9.6)	—	374	10.8%	0.77 (0.07)	—
Type of housing at recruitment	3457	—	—	0.18	3453	—	—	0.51	3452	—	—	0.47
Public	1448	41.9%	21.1 (3.6)	—	1445	41.8%	72.5 (9.4)	—	1445	41.9%	0.77 (0.07)	—
Subsidized home ownership scheme	545	15.8%	20.8 (3.6)	—	544	15.8%	72.0 (9.2)	—	544	15.8%	0.77 (0.06)	—
Private	1359	39.3%	20.9 (3.4)	—	1359	39.4%	72.3 (9.1)	—	1358	39.3%	0.77 (0.06)	—
Unknown	105	3.0%	20.5 (3.0)	—	105	3.0%	71.2 (7.5)	—	105	3.0%	0.76 (0.05)	—

*Two-side *P*-value from independent t-test or analysis of variance (ANOVA).

Table 2 shows ALT was positively associated with BMI, WC, and WHR adjusted for potential confounders. ALP was negatively associated with BMI, WC, and WHR. The associations of ALP with BMI, WC, and WHR differed by sex, with the inverse associations only evident in boys.

**Table 2:** Adjusted associations of liver enzymes (ALT and ALP) with adiposity indices (BMI, WC, and WHR) at ~17.5 years in the Hong Kong’s “Children of 1997” birth cohort, Hong Kong, China.

Liver enzyme	Outcome	Sex-adjusted		Sex interaction	Boys		Girls	
		Beta	95% CI	p-value	Beta	95% CI	Beta	95% CI
ALT (IU/L)	BMI (kg/m^2^)	0.10	0.09 to 0.11	0.19	0.10	0.09 to 0.12	0.09	0.07 to 0.11
	WC (cm)	0.25	0.23 to 0.28	0.04	0.27	0.24 to 0.30	0.21	0.17 to 0.26
	WHR	0.0013	0.0012 to 0.0015	0.08	0.0014	0.0012 to 0.0016	0.0011	0.0008 to 0.0014
ALP (IU/L)	BMI (kg/m^2^)	−0.018	−0.024 to −0.012	<0.001	−0.024	−0.031 to −0.017	0.006	−0.006 to 0.018
	WC (cm)	−0.03	−0.05 to −0.02	<0.001	−0.05	−0.07 to −0.03	0.04	0.01 to 0.07
	WHR	−0.0002	−0.0003 to −0.0001	0.002	−0.0002	−0.0004 to −0.0001	0.0001	−0.0001 to 0.0003

ALT: alanine aminotransferase; ALP: alkaline phosphatase.

BMI: body mass index; WC: waist circumference; WHR: waist-hip ratio.

Adjustment: household income, highest parental education, type of housing, highest parental occupation, second-hand and maternal smoking.

### Mendelian randomization

#### Genetic variants

In total, 4 single nucleotide polymorphisms (SNPs) independently predicting ALT, 14 SNPs independently predicting ALP, and 26 SNPs independently predicting GGT at genome-wide significance were obtained (Supplemental Table S2)^14^. All the palindromic SNPs were aligned based on effect allele frequency (Supplemental Table S3), except for rs2073398 (*GGT1, GGTLC*2), predicting GGT, which was replaced by rs5751901 (R^2^ = 0.95) for GIANTUKB. Rs6834314 (*HSD17B13, MAPK10*) predicting ALT and rs944002 (*EXOC3L4*) predicting GGT were replaced in the genome-wide association study (GWAS) Anthropometric 2015 Waist by rs13102451 (R^2^ = 1.00) and rs2297067 (R^2^ = 0.98). Two SNPs, rs516246 (*FUT*2) and rs8038465 (*CD276*) predicting GGT had rather different allele distributions for GGT and adiposity indices in GIANT (GWAS Anthropometric 2015 BMI and the GWAS Anthropometric 2015 Waist)^15,^^16^. They were dropped in a sensitivity analysis separately. No proxy SNP (R^2^ > 0.9) of rs516246 could be found in GIANTUKB. (Supplemental Table S4).

Of the 4 SNPs predicting ALT, rs2954021 (*TRIB1*) predicted both ALT and ALP, and rs738409 (*PNPLA3*) is highly associated with NAFLD. Of the 14 SNPs predicting ALP, rs281377 (*FUT2*) is highly associated with resting metabolic rate; rs579459 is located in the *ABO* gene. Of the 26 SNPs predicting GGT, rs516246 (*FUT2*) is associated with obesity-related traits; rs1260326 (*GCKR*) is associated with Crohn’s disease which might be associated with adiposity (Supplemental Table S2). The *F* statistics and variance explained (r^2^) were 15 and 0.001 for ALT, 158 and 0.035 for ALP, and 45 and 0.019 for GGT. As such the MR study had 80% power with 5% alpha to detect a difference of 0.11, 0.02, and 0.02 in BMI effect size for ALT, ALP, and GGT respectively.

#### Genetically instrumented ALT with BMI, WC, and WHR

Genetically instrumented ALT was negatively associated with BMI using inverse variance weighting (IVW), which was also evident using a weighted median (WM) and after excluding the potentially pleiotropic SNPs. The negative association was more obvious for women. ALT was not associated with WC or WHR using any method with and without potentially pleiotropic SNPs. Few MR-Egger intercepts differed from the null, giving little indication of pleiotropy. Heterogeneity was almost absent after the pleiotropic SNP were excluded (Tables 3–5).

**Table 3:** Estimates of the effect of genetically instrumented liver enzymes (ALT, ALP, and GGT) (per 100% change in concentration) on BMI (standard deviation) using Mendelian randomization with different methodological approaches with and without potentially pleiotropic SNPs.

Liver enzyme	Method	SNP	Men and women together using All-GIANTUKB				Men using GWAS Anthropometric 2015 BMI				Women using GWAS Anthropometric 2015 BMI			
			Beta	95% CI	MR-Egger	I^2^	Beta	95% CI	MR-Egger	I^2^	Beta	95% CI	MR-Egger	I^2^
					Intercept p-value	(p-value)			Intercept p-value	(p-value)			Intercept p-value	(p-value)
ALT	IVW	4	−0.17	−0.33 to −0.01	—	84.6% (<0.001)	−0.13	−0.39 to 0.13	—	60.6% (0.05)	−0.19	−0.34 to −0.04	—	0.0% (0.76)
		3	−0.14	−0.20 to −0.07	—	0.0% (<0.64)	−0.08	−0.24 to 0.09	—	0.0% (0.39)	−0.18	−0.34 to −0.03	—	0.0% (0.59)
		2	−0.17	−0.29 to −0.05	—	—	−0.24	−0.56 to 0.07	—	—	−0.19	−0.48 to 0.10	—	—
	WM	4	−0.13	−0.20 to −0.05	—	—	−0.06	−0.25 to 0.12	—	—	−0.19	−0.36 to −0.02	—	—
		3	−0.12	−0.20 to −0.05	—	—	−0.05	−0.23 to 0.13	—	—	−0.18	−0.36 to −0.01	—	—
	MR-Egger	4	0.02	−0.25 to 0.30	0.11	—	0.24	−0.09 to 0.58	0.01	—	−0.16	−0.48 to 0.15	0.85	—
		3	−0.09	−0.25 to 0.06	0.54	—	0.14	−0.26 to 0.53	0.25	—	−0.19	−0.57 to 0.19	0.96	—
ALP	IVW	14	−0.07	−0.18 to 0.04	—	88.5% (<0.001)	0.03	−0.10 to 0.17	—	60.4% (0.002)	−0.08	−0.21 to 0.04	—	58.7% (0.003)
		13	−0.06	−0.16 to 0.05	—	86.2% (<0.001)	0.05	−0.07 to 0.17	—	51.6% (0.02)	−0.08	−0.21 to 0.05	—	61.3% (0.002)
		11	−0.07	−0.23 to 0.09	—	88.3% (<0.001)	−0.02	−0.22 to 0.18	—	55.7% (0.01)	−0.17	−0.33 to −0.01	—	34.6% (0.12)
	WM	14	−0.05	−0.11 to −0.001	—	—	0.07	−0.03 to 0.18	—	—	−0.06	−0.16 to 0.04	—	—
		13	−0.05	−0.10 to −0.001	—	—	0.07	−0.03 to 0.18	—	—	−0.06	−0.16 to 0.04	—	—
		11	−0.07	−0.18 to 0.04	—	—	−0.12	−0.33 to 0.09	—	—	−0.11	−0.29 to 0.07	—	—
	MR-Egger	14	0.03	−0.17 to 0.22	0.23	—	0.15	−0.06 to 0.37	0.18	—	−0.04	−0.26 to 0.18	0.63	—
		13	−0.001	−0.19 to 0.19	0.50	—	0.12	−0.09 to 0.33	0.39	—	−0.05	−0.28 to 0.19	0.74	—
		11	0.28	−0.24 to 0.79	0.17	—	0.21	−0.49 to 0.92	0.50	—	0.01	−0.54 to 0.56	0.51	—
GGT^*^	IVW	26/25	0.04	−0.01 to 0.08	—	85.6% (<0.001)	0.01	−0.05 to 0.07	—	40.7% (0.02)	0.001	−0.06 to 0.07	—	56.0% (<0.001)
		23/24	0.04	−0.003 to 0.09	—	83.2% (<0.001)	0.02	−0.03 to 0.07	—	12.2% (0.29)	0.02	−0.05 to 0.08	—	50.4% (0.003)
	WM	26/25	0.03	0.01 to 0.06	—	—	0.02	−0.05 to 0.09	—	—	−0.01	−0.07 to 0.06	—	—
		23/24	0.03	0.01 to 0.06	—	—	0.02	−0.05 to 0.09	—	—	−0.01	−0.07 to 0.06	—	—
	MR-Egger	26/25	0.06	−0.05 to 0.17	0.65	—	0.05	−0.09 to 0.19	0.47	—	0.04	−0.11 to 0.19	0.56	—
		23/24	0.05	−0.06 to 0.15	0.97	—	0.03	−0.10 to 0.15	0.96	—	−0.01	−0.16 to 0.14	0.67	—

*Rs516246 (*FUT2*) predicting GGT is not available in GIANTUKB, 25 SNPs remained.

Excluded SNPs predicting ALT: rs2954021 (*TRIB1*) when SNP = 3, excluded rs738409 (*PNPLA3*) in addition when SNP = 2; excluded SNPs predicting ALP: rs2954021 (*TRIB1*), when SNP = 13; excluded rs281377 (*FUT2*) and rs579459 (*ABO*) in addition when SNP = 11; excluded SNPs predicting GGT: rs516246 (*FUT2*), rs1260326 (*C2orf16, GCKR*), and rs8038465 (*CD276*) when SNP = 23 from GWAS Anthropometric 2015 BMI projects, excluding SNPs: rs516246 (*FUT2*) and rs1260326 (*C2orf16, GCKR*) when SNP = 24 from GIANTUKB.

SNP: single nucleotide polymorphism; ALT: alanine aminotransferase; ALP: alkaline phosphatase; GGT: gamma glutamyltransferase; BMI: body mass index; IVW: inverse variance weighted; WM: weighted median.

**Table 4:** Estimates of the effect of genetically instrumented liver enzymes (ALT, ALP, and GGT) (per 100% change in concentration) on WC (standard deviation) using Mendelian randomization with different methodological approaches with and without potentially pleiotropic SNPs.

Liver enzyme	Method	SNPs	All				Men				Women			
			Beta	95% CI	MR-Egger	I^2^	Beta	95% CI	MR-Egger	I^2^	Beta	95% CI	MR-Egger	I^2^
					Intercept p- value	(p-value)			Intercept p-value	(p-value)			Intercept p-value	(p-value)
ALT	IVW	4	−0.06	−0.23 to 0.12	—	34.5% (0.21)	0.0001	−0.35 to 0.35	—	66.4% (0.03)	−0.08	−0.26 to 0.10	—	9.3% (0.35)
		3	−0.03	−0.18 to 0.11	—	40.2% (0.19)	0.08	−0.13 to 0.29	—	0.0% (0.66)	−0.1	−0.28 to 0.08	—	25.8% (0.26)
		2	−0.03	−0.29 to 0.24	—	—	0.08	−0.31 to 0.46	—	—	−0.06	−0.39 to 0.27	—	—
	WM	4	−0.06	−0.22 to 0.10	—	—	0.06	−0.17 to 0.29	-	—	−0.1	−0.29 to 0.10	—	—
		3	−0.04	−0.21 to 0.12	—	—	0.08	−0.15 to 0.31	—	—	−0.12	−0.32 to 0.08	—	—
	MR-Egger	4	0.01	−0.41 to 0.44	0.72	—	0.35	−0.34 to 1.05	0.26	—	−0.23	−0.62 to 0.17	0.42	—
		3	−0.08	−0.70 to 0.54	0.87	—	0.06	−0.44 to 0.56	0.93	—	−0.2	−0.86 to 0.46	0.75	—
ALP	IVW	14	−0.02	−0.16 to 0.11	—	72.6% (<0.001)	−0.02	−0.18 to 0.14	—	62.8% (<0.001)	−0.03	−0.15 to 0.10	—	51.0% (0.01)
		13	−0.02	−0.15 to 0.12	—	73.9% (<0.001)	−0.004	−0.15 to 0.15	—	56.5% (0.006)	−0.03	−0.16 to 0.10	—	54.2% (0.01)
		11	−0.13	−0.33 to 0.08	—	70.8% (<0.001)	−0.09	−0.33 to 0.15	—	55.7% (0.01)	−0.15	−0.34 to 0.04	—	45.0% (0.05)
	WM	14	0.01	−0.08 to 0.10	—	—	0.002	−0.12 to 0.12	—	—	0.03	−0.09 to 0.14	—	—
		13	0.02	−0.07 to 0.10	—	—	0.01	−0.11 to 0.13	—	—	0.02	−0.09 to 0.14	—	—
		11	−0.13	−0.32 to 0.06	—	—	−0.19	−0.43 to 0.06	—	—	−0.06	−0.28 to 0.15	—	—
	MR-Egger	14	0.1	−0.13 to 0.32	0.20	—	0.14	−0.13 to 0.40	0.15	—	0.06	−0.16 to 0.28	0.33	—
		13	0.09	−0.14 to 0.32	0.27	—	0.1	−0.15 to 0.36	0.31	—	0.08	−0.15 to 0.30	0.26	—
		11	0.27	−0.43 to 0.97	0.25	—	0.47	−0.32 to 1.27	0.14	—	0.12	−0.53 to 0.78	0.39	—
GGT	IVW	26	−0.01	−0.05 to 0.03	—	14.2% (0.26)	−0.04	−0.10 to 0.03	—	24.8% (0.12)	0.01	−0.05 to 0.07	—	35.7% (0.04)
		23	0.003	−0.04 to 0.04	—	0.0% (0.56)	−0.02	−0.07 to 0.04	—	1.4% (0.44)	0.02	−0.04 to 0.08	—	30.8% (0.08)
	WM	26	−0.04	−0.10 to 0.02	—	—	−0.01	−0.09 to 0.06	—	—	−0.002	−0.08 to 0.07	—	—
		23	−0.04	−0.09 to 0.02	—	—	−0.01	−0.09 to 0.06	—	—	−0.002	−0.08 to 0.07	—	—
	MR-Egger	26	0.05	−0.05 to 0.14	0.18	—	0.05	−0.10 to 0.19	0.23	—	0.05	−0.09 to 0.19	0.50	—
		23	0.01	−0.09 to 0.10	0.91	—	0.02	−0.12 to 0.16	0.57	—	0.01	−0.14 to 0.15	0.84	—

Excluded SNPs predicting ALT: rs2954021 (*TRIB1*) when SNP = 3, excluded rs738409 (*PNPLA3*) in addition when SNP = 2; excluded SNPs predicting ALP: rs2954021 (*TRIB1*), when SNP = 13; excluded rs281377 (*FUT2*) and rs579459 (*ABO*) in addition when SNP = 11; excluded SNPs predicting GGT: rs516246 (*FUT2*), rs1260326 (*C2orf16, GCKR*), and rs8038465 (*CD276*) when SNP = 23.

SNP: single nucleotide polymorphism; ALT: alanine aminotransferase; ALP: alkaline phosphatase; GGT: gamma glutamyltransferase; WC: waist circumference; IVW: inverse variance weighted; WM: weighted median.

**Table 5:** Estimates of the effect of genetically instrumented liver enzymes (ALT, ALP, and GGT) (per 100% change in concentration) on WHR (standard deviation) using Mendelian randomization with different methodological approaches with and without potentially pleiotropic SNPs.

Liver enzyme	Method	SNP	All				Men				Women			
			Beta	95% CI	MR-Egger	I^2^	Beta	95% CI	MR-Egger	I^2^	Beta	95% CI	MR-Egger	I^2^
					Intercept p-value	(p-value)			Intercept p-value	(p-value)			Intercept p-value	(p-value)
ALT	IVW	4	0.04	−0.09 to 0.18	—	0.0% (0.73)	0.09	−0.13 to 0.30	—	10.2% (0.34)	0.02	−0.24 to 0.27	—	56.2% (0.08)
		3	0.03	−0.11 to 0.17	—	0.0% (0.81)	0.14	−0.08 to 0.35	—	0.0% (0.83)	−0.04	−0.22 to 0.13	—	0.0% (0.81)
		2	−0.02	−0.28 to 0.24	—	—	0.15	−0.27 to 0.55	—	—	−0.12	−0.45 to 0.20	—	—
	WM	4	0.05	−0.10 to 0.20	—	—	0.12	−0.11 to 0.35	—	—	−0.02	−0.21 to 0.16	—	—
		3	0.04	−0.11 to 0.19	—	—	0.13	−0.10 to 0.36	—	—	−0.03	−0.21 to 0.16	—	—
	MR-Egger	4	−0.004	−0.29 to 0.28	0.70	—	0.29	−0.16 to 0.74	0.31	—	−0.19	−0.74 to 0.36	0.40	—
		3	0.08	−0.25 to 0.41	0.73	—	0.1	−0.41 to 0.61	0.88	—	0.06	−0.35 to 0.47	0.59	—
ALP	IVW	14	−0.03	−0.16 to 0.10	—	73.6% (<0.001)	−0.11	−0.26 to 0.05	—	60.0% (0.002)	0.04	−0.11 to 0.19	—	66.2% (<0.001)
		13	−0.03	−0.17 to 0.10	—	74.9% (<0.001)	−0.1	−0.26 to 0.06	—	61.4% (0.002)	0.02	−0.12 to 0.17	—	63.4% (0.001)
		11	−0.11	−0.34 to 0.12	—	77.5% (<0.001)	−0.08	−0.36 to 0.20	—	66.7% (<0.001)	−0.11	−0.32 to 0.11	—	57.9% (0.008)
	WM	14	0.02	−0.06 to 0.10	—	—	−0.12	−0.25 to 0.00	—	—	0.12	0.01 to 0.24	—	—
		13	0.02	−0.06 to 0.10	—	—	−0.12	−0.24 to −0.001	—	—	0.12	0.01 to 0.23	—	—
		11	−0.002	−0.17 to 0.17	—	—	0.02	−0.23 to 0.27	—	—	0.02	−0.17 to 0.21	—	—
	MR-Egger	14	0.08	−0.14 to 0.30	0.23	—	−0.04	−0.31 to 0.23	0.54	—	0.17	−0.09 to 0.43	0.21	—
		13	0.1	−0.12 to 0.32	0.14	—	−0.06	−0.34 to 0.23	0.69	—	0.22	−0.01 to 0.45	0.04	—
		11	0.35	−0.42 to 1.13	0.22	—	0.7	−0.18 to 1.57	0.07	—	0.11	−0.64 to 0.86	0.56	—
GGT	IVW	26	0.03	−0.01 to 0.07	—	13.6% (0.27)	0.02	−0.03 to 0.08	—	0.0% (0.88)	0.04	−0.02 to 0.10	—	37.8% (0.03)
		23	0.03	−0.02 to 0.07	—	19.8% (0.20)	0.03	−0.03 to 0.09	—	0.0% (0.85)	0.03	−0.03 to 0.10	—	37.4% (0.04)
	WM	26	−0.01	−0.06 to 0.05	—	-	0.02	−0.06 to 0.10	—	—	−0.01	−0.08 to 0.07	—	—
		23	−0.01	−0.07 to 0.05	—	—	0.02	−0.06 to 0.10	—	—	−0.02	−0.09 to 0.06	—	—
	MR-Egger	26	0.03	−0.06 to 0.13	0.95	—	0.08	−0.05 to 0.21	0.31	—	0.01	−0.13 to 0.15	0.64	-
		23	0.04	−0.07 to 0.15	0.84	—	0.07	−0.06 to 0.21	0.50	—	0.03	−0.12 to 0.18	0.94	—

Excluded SNPs predicting ALT: rs2954021 (*TRIB1*) when SNP = 3, excluded rs738409 (*PNPLA3*) in addition when SNP = 2; excluded SNPs predicting ALP: rs2954021 (*TRIB1*), when SNP = 13; excluded rs281377 (*FUT2*) and rs579459 (*ABO*) in addition when SNP = 11; excluded SNPs predicting GGT: rs516246 (*FUT2*), rs1260326 (*C2orf16, GCKR*), and rs8038465 (*CD276*) when SNP = 23.

SNP: single nucleotide polymorphism; ALT: alanine aminotransferase; ALP: alkaline phosphatase; GGT: gamma glutamyltransferase; WHR: waist-hip ratio; IVW: inverse variance weighted; WM: weighted median.

#### Genetically instrumented ALP and GGT with BMI, WC, and WHR

Genetically instrumented ALP and GGT were not clearly associated with BMI, WC, or WHR using any method with and without potentially pleiotropic SNPs. Overall, there was no evidence of pleiotropy based on the null values of the MR-Egger intercepts. Heterogeneity was most evident for ALP (Tables 3–5).

## Discussion

This novel study used two different approaches, an observational study and an MR study, with different data sources, assumptions, and different unrelated sources of bias to assess the role of liver enzymes in adiposity. We found the clearest evidence for ALT being inversely associated with BMI, perhaps particularly among women.

We used an observational design to assess the association of liver function, indicated by liver enzymes, with adiposity indices in young people aged 17.5 years and an MR design in adults. However, limitations exist in both study designs. First, liver enzymes represent different aspects of liver function: ALT is a marker of hepatocyte integrity, which is relatively more specific for liver pathology than other indices. ALP and GGT are markers of cholestasis. ALP is not liver specific and also originates from other tissues especially from bone. As such, ALT, ALP, and GGT may not completely or only represent liver function^18^. Second, the two study designs may not be completely comparable. Specifically, the observational study pertains to Chinese, while the MR study mainly pertains to people of European ancestry because a suitably large GWAS of Chinese people is not publicly available. However, causes are usually consistent although not relevant in all contexts^19^. Finally, the two study designs have contrasting limitations.

The conventional observational study is open to residual confounding by factors such as diet, lifestyle, and physical activity, which are hard to measure precisely and eliminate, although smoking is rare, alcohol use is low, and adiposity is not strongly associated with SEP in Hong Kong^20,21^, which may reduce confounding. However, it is difficult to disentangle correlated factors reliably in such observational studies. ALT was also lower than 10 IU/L (n = 254) for 7.3% of the participants in “Children of 1997” and was fixed at 5 IU/L, which was unlikely to affect the estimates, because it was only below the limit of detection for a relatively small proportion of observations. Follow-up in “Children of 1997” was incomplete, however, no major differences were found between the participants with and without adiposity indices (Supplemental Table S1). As such, selection bias from loss-to-follow-up is unlikely.

MR studies are more robust to confounding than conventional observational studies but have strong assumptions. Specifically MR studies rely on the assumptions that the genetic instruments predict the exposure reliably, are independent of confounders of the exposure-outcome association, and are only associated with the outcome via the exposure. The *F* statistics were all larger than 10, which reduces the risk of weak instrument bias. Pleiotropic effects are possible, but estimates were similar after excluding potentially pleiotropic SNPs, such as rs738409 (*PNPLA3*) predicting ALT and MR-Egger did not provide statistical evidence of pleiotropy. Although some of the *I*^*2*^ were large, after excluding potential pleiotropic SNPs, in most cases, the *I*^*2*^ became smaller. Estimates for ALP showed some heterogeneity although the MR-Egger regression did not show directional pleiotropy. The GWAS for liver enzymes overlapped slightly (~17%) with the GWAS of adiposity indices from the GIANT consortium but is unlikely to cause bias. We assessed sex differences on the assumption that genetic predictors of liver function are similar for women and men, which we could not test empirically. Finally, MR provides an estimate of the effect of life time exposure rather than indicating the exact size of the corresponding intervention, as such it indicates an etiological pathway.

Observationally, the positive associations of ALT with BMI, WC, and WHR are consistent with most of the previous observational studies in both adolescents and adults^22–26^. The negative associations of ALP with adiposity are consistent with a previous study among Australian adolescents^27^, but not with all studies^28^, although few such studies have been conducted. However, some estimates differed between the observational and MR designs, probably because of the difficulty of distinguishing correlated measures of liver function, the possibility of confounding, and/or observational studies not reflecting life-long effects.

To our knowledge, only one small MR study has assessed the association of liver function with adiposity, and found no association of ALT with BMI^7^. One possible explanation for ALT potentially reducing BMI, but not WHR or WC, is that ALT, acting via sex hormones, reduces muscle mass rather than or as well as fat mass. ALT reducing muscle mass would be consistent with ALT increasing the risk of diabetes^3^, because low muscle mass is a potential cause of diabetes^29^.

Overall, this study suggests that ALT reduces BMI. To further clarify the role of liver function in metabolic conditions whether ALT reduces specifically muscle mass, and thereby causes diabetes should be investigated, because it would mean that muscle mass could be an attractive target of intervention to prevent diabetes.

## Methods

### The “Children of 1997” birth cohort

The “Children of 1997” birth cohort is a population-representative Chinese birth cohort (n = 8,327) which recruited 88% of all births in Hong Kong in April and May 1997^30^. The study was originally established to assess the effects of second-hand smoke exposure and breastfeeding on health services utilization in the first 18 months of life. Recruitment was conducted at the first postnatal visit to the Maternal and Child Health Centers in Hong Kong. Parents of all newborns were encouraged to attend to obtain free preventive care and vaccinations for their child. Parental and infant characteristics were obtained from a self-administered questionnaire in Chinese at recruitment and subsequent routine visits. In 2007, contact was re-established followed by three postal/telephone questionnaire surveys. From 2013 to 2016 a Biobank Clinical follow-up at 16–18 years was conducted, when liver enzymes were assessed. As a compromise between cost and comprehensiveness, liver enzymes were assessed from plasma ALT and plasma ALP analyzed using the Roche Cobas C8000 System, a discrete photometric chemistry analyzer, with International Federation of Clinical Chemistry standardized method with pyridoxal phosphate and substrates of L-alanine and 2-oxoglutarate for ALT, and an optimized substrate concentration and 2-amino-2-methyl-1-propanol as buffer plus the cations magnesium and zinc for ALP. These analyses were conducted at an accredited laboratory serving a teaching hospital in Hong Kong. Height, weight, and waist and hip circumference were measured using standard protocols.

### Children of 1997

#### Exposure - liver enzymes

Liver function at ~17.5 years was assessed from plasma ALT (IU/L) and plasma ALP (IU/L).

#### Outcome - Adiposity

Adiposity was assessed from BMI (kg/m^2^), WC (cm), and WHR, which represent different aspects of adiposity. Although these are not completely normally distributed, we present them in natural units for ease of interpretation, given interpretation was similar using a gamma distribution.

### Mendelian randomization

#### Genetic associations with liver enzymes

SNPs associated with plasma log transformed ALT, ALP, and GGT at genome-wide significance (p-value < 5 × 10^−8^) adjusted for age and sex were obtained from the largest available GWAS of plasma levels of liver enzymes comprising 61,089 adults (~86% European, mean age 52.8 years, 50.6% women)^14^. For SNPs in linkage disequilibrium (R^2^ > 0.01), we retained SNPs with the lowest p-value using the *Clumping* function of MR-Base (*TwoSampleMR*) R package, based on the 1000 Genomes catalog^31^. Whether any of the selected SNPs was related to adiposity directly rather than through liver enzymes (pleiotropic effects) was assessed from their known phenotypes obtained from comprehensive curated genotype to phenotype cross-references, i.e., Ensembl (http://www.ensembl.org/index.html) and the GWAS Catalog (https://www.ebi.ac.uk/gwas/). We also identified SNPs from highly pleiotropic genes, such as *ABO* and *GCKR*, whose full functionality is not yet clearly understood.

#### Genetic associations with adiposity

Overall genetic associations with BMI (SD units) were obtained from 2018 GIANT and UK Biobank meta-analysis (GIANTUKB) (n = 681,275)^17^, a meta-analysis of the GIANT GWAS Anthropometric 2015 BMI^15^ (mean age 56.0 years, 53.8% women, 95% European) with a newly conducted GWAS of UK Biobank (100% European). The sample overlap is negligible between these two GWAS^17^. Sex-specific genetic associations with BMI were from the GIANT GWAS Anthropometric 2015 BMI^15^ (n = 339,224, mean age 56.0 years, 53.8% women, 95% European). Overall and sex-specific genetic associations with WC (SD units) and WHR (SD units) were obtained from the GIANT GWAS Anthropometric 2015 Waist^16^ (n = 224,459, mean age 54.5 years, 54.6% women, 63.6% European). The GIANTUKB adjusted for age, sex, 10 principal components, recruitment centre, and genotyping batches^17^. The GIANT (GWAS Anthropometric 2015 BMI and the GWAS Anthropometric 2015 Waist) adjusted for age, age-squared, study-specific covariates in a linear model^15,16^.

### Statistical analyses

In the “Children of 1997” birth cohort, baseline characteristics of cohort participants who were included and excluded were compared using Cohen effect sizes^32^. Cohen effect sizes indicate the magnitude of the difference independent of sample size. They are usually categorized as 0.10 for small, 0.30 for medium, and 0.50 for large when considering categorical variables^32^. The associations of adiposity indices with potential confounders were assessed using independent t-test or analysis of variance (ANOVA).

We assessed the associations of liver enzymes with adiposity indices adjusted for potential confounders, i.e., household income, highest parental education, type of housing, highest parental occupation, second-hand and maternal smoking, and sex, using multivariable linear regression. We also assessed whether associations differed by sex from the relevant interaction terms.

In the Mendelian randomization study, the strength of the genetic instruments was indicated by the *F*-statistic^33^. A higher *F*-statistic indicates lower risk of weak instrument bias^33^. We aligned SNPs for exposure and outcome on allele and effect allele frequency to ensure all SNPs, in particular palindromic SNPs, were aligned correctly. SNPs that could not be unequivocally aligned were replaced by proxies or dropped. SNPs predicting liver enzymes that were not available for adiposity indices were replaced by highly correlated proxies (R^2^ > 0.9). Potential proxy SNPs were obtained from the GWAS^14^ and their correlations with other SNPs were obtained using LDlink^34,35^.

Unconfounded estimates of the effects of liver enzymes on adiposity indices overall and by sex were obtained by meta-analyzing SNP-specific Wald estimates (SNP-outcome association divided by SNP-exposure association) using IVW with random effects for 4+ SNPs, which assumes that balanced pleiotropy, and with fixed effects for 3 SNPs or fewer. We repeated the analysis excluding pleiotropic SNPs that might be associated with the relevant outcome directly rather than via liver enzymes. As sensitivity analyses, WM and MR-Egger regression were used. The WM may generate correct estimates when >50% of weight is contributed by valid SNPs^36^. MR-Egger generates correct estimates even when all the SNPs are invalid instruments as long as the instrument strength independent of direct effect assumption is satisfied^37^. A non-null intercept from MR-Egger indicates potential directional pleiotropy and invalid IVW estimates^36^. Heterogeneity was assessed using the *I*^2^ statistic^37^. Power calculations were performed using the approximation that the sample size for an Mendelian randomization equates to that of the same regression analysis with the sample size divided by the r^2^ for genetic variant on exposure^38^.

All statistical analyses were conducted using R version 3.4.2 (R Foundation for Statistical Computing, Vienna, Austria). The R package MendelianRandomization^39^ was used to generate the estimates.

### Ethics approval and informed consent

Ethical approval for the study, including comprehensive health related analyses, were obtained from Institutional Review Board of the University of Hong Kong/Hospital Authority Hong Kong West Cluster (HKU/HA HKW IRB). Informed written consent was obtained from the parents/guardians or participant if 18 years or older before participation in the Biobank Clinical Follow-up.

The MR study only uses published or publicly-available data. No original data were collected for the MR study. Ethical approval for each of the studies included in the investigation can be found in the original publications (including informed consent from each participant).

## Supplementary information

Dataset 1

## Data Availability

Data are available upon request from the “Children of 1997” data access committee: aprmay97@hku.hk. The volume and complexity of the data collected preclude public data deposition, because the participants could be identifiable from such extensive data which would comprise participant privacy. Data of the MR study are publicly available summary data.
