# Supplementary material for: The effect of liver enzymes on adiposity: a Mendelian randomization study
Source: Sci Rep. 2019 Nov 14;9:16792. doi: 10.1038/s41598-019-52489-8 (PMC6856156; doi:10.1038/s41598-019-52489-8)
Supplement: Supplementary file 1 — Dataset 1 [file 41598_2019_52489_MOESM1_ESM.docx]

**The effect of liver enzymes on adiposity: a Mendelian randomization study**

Jun Xi Liu^1^, Shiu Lun Au Yeung^1^, Man Ki Kwok^1^, June Yue Yan Leung^1^, Shi Lin Lin^1^, Lai Ling Hui^1,2^, Gabriel Matthew Leung^1^, C. Mary Schooling^1,3*^

^1^School of Public Health, Li Ka Shing Faculty of Medicine, The University of Hong Kong, Hong Kong SAR, China

^2^Department of Paediatrics, Faculty of Medicine, the Chinese University of Hong Kong, Hong Kong SAR, China

^3^City University of New York Graduate School of Public Health and Health Policy, New York, NY, USA

***Corresponding author:**

Dr. C Mary Schooling

School of Public Health

Li Ka Shing Faculty of Medicine

The University of Hong Kong

1/F, Patrick Manson Building (North Wing), 7 Sassoon Road,

Hong Kong SAR, China

Telephone: (852) 3917 6732

Fax: (852) 3520 1945

E-mail: [cms1@hku.hk](mailto:cms1@hku.hk)

**Table legends:**

Supplemental Table S1. Baseline characteristics of the participants who were included (n=3458) and excluded (n=4869) in the analyses of the Hong Kong’s “Children of 1997” birth cohort, Hong Kong, China, 1997 to 2016.

Supplemental Table S2. Single nucleotide polymorphisms (SNPs) with potential pleiotropic effects from Ensembl and from GWAS Catalog.

Supplemental Table S3. Characteristics of palindromic single nucleotide polymorphisms (SNPs) in the exposure and outcome genome-wide association studies (GWAS).

Supplemental Table S4. Characteristics of unequivocally aligned single nucleotide polymorphisms (SNPs) in the exposure and outcome genome-wide association study (GWAS).

Supplemental Table S1. Baseline characteristics of the participants who were included (n=3458) and excluded (n=4869) in the analyses of the Hong Kong’s “Children of 1997” birth cohort, Hong Kong, China, 1997 to 2016.

| Characteristics | | Included (n=3458) | | Excluded (n=4869) | | Cohen effect size Φ |
| --- | --- | --- | --- | --- | --- | --- |
|  |  |  |  |  |  |  |
|  |  | n | % | n | % |  |
| Sex | | 3458 | - | 4869 | - | 0.08 |
|  | Female | 1717 | 49.70% | 2196 | 45.10% | - |
|  | Male | 1741 | 50.30% | 2608 | 53.60% | - |
|  | Unknown | 0 | - | 65 | 1.30% | - |
| Second-hand and maternal smoking exposure | | 3458 | - | 4869 | - | 0.09 |
|  | None | 943 | 27.30% | 1232 | 25.30% | - |
|  | Prenatal second-hand smoking | 1280 | 37.00% | 1520 | 31.20% | - |
|  | Postnatal second-hand smoking | 962 | 27.80% | 1492 | 30.60% | - |
|  | Maternal smoking | 128 | 3.70% | 275 | 5.60% | - |
|  | Unknown | 145 | 4.20% | 350 | 7.20% | - |
| Highest parental education levels | | 3458 | - | 4869 | - | 0.12 |
|  | Grade<=9 | 991 | 28.70% | 1476 | 30.30% | - |
|  | Grades 10-11 | 1490 | 43.10% | 1957 | 40.20% | - |
|  | Grades>=12 | 961 | 27.80% | 1222 | 25.10% | - |
|  | Unknown | 16 | 0.50% | 214 | 4.40% | - |
| Highest parental occupation | | 3458 | - | 4869 | - | 0.07 |
|  | Ⅰ(unskilled) | 99 | 2.90% | 140 | 2.90% | - |
|  | Ⅱ(semiskilled) | 285 | 8.20% | 441 | 9.10% | - |
|  | Ⅲ (semiskilled) | 504 | 14.60% | 711 | 14.60% | - |
|  | Ⅲ (nonmanual skilled) | 879 | 25.40% | 1167 | 24.00% | - |
|  | Ⅳ (managerial) | 440 | 12.70% | 682 | 14.00% | - |
|  | Ⅴ(professional) | 797 | 23.00% | 917 | 18.80% | - |
|  | Unknown | 454 | 13.10% | 811 | 16.70% | - |
| Household income per head at recruitment | | 3458 | - | 4869 | - | 0.07 |
|  | First quintile | 573 | 16.60% | 878 | 18.00% | - |
|  | Second quintile | 616 | 17.80% | 868 | 17.80% | - |
|  | Third quintile | 618 | 17.90% | 811 | 16.70% | - |
|  | Fourth quintile | 631 | 18.20% | 788 | 16.20% | - |
|  | Fifth quintile | 646 | 18.70% | 794 | 16.30% | - |
|  | Unknown | 374 | 10.80% | 730 | 15.00% | - |
| Type of housing at recruitment | | 3458 | - | 4869 | - | 0.08 |
|  | Public | 1448 | 41.90% | 2128 | 43.70% | - |
|  | Subsidized home ownership scheme | 545 | 15.80% | 580 | 11.90% | - |
|  | Private | 1360 | 39.30% | 1885 | 38.70% | - |
|  | Unknown | 105 | 3.00% | 276 | 5.70% | - |

Φ Cohen effect sizes are usually categorized into 3 levels, Chi-square tests for categorical variables: 0.10 for small. 0.30 for medium, 0.50 for large. For categorical variables, Cohen’s w effect size is calculated as $w=\sqrt{\sum{({p0-p1)}^{2}}/{p0}}$, where p0 is the proportion in given by the null hypothesis and p1 is the proportion given the alternative hypothesis; $w=\sqrt{{\chi2}/N}$ where N is the total count of the included and excluded participants.

Supplemental Table S2. Single nucleotide polymorphisms (SNPs) with potential pleiotropic effects from Ensembl and from GWAS Catalog.

| Liver Enzyme | SNPs | Location | Gene nearby | Phenotype, disease and trait -Ensembl | Phenotype, disease and trait - GWAS Catalog | Potential pleiotropy |
| --- | --- | --- | --- | --- | --- | --- |
| ALT | rs10883437 | 10q24 | *CPN1* | - | - | - |
| ALT | rs2954021 | 8q24 | *TRIB1* | HDL, LDL, TC, ALP, Lymphocyte percentage of white cells, Neutrophil percentage of white cells, Response to fenofibrate (triglyceride levels) | Triglyceride levels, ALP, LDL | + |
| ALT | rs6834314 | 4q22 | *HSD17B13, MAPK10* | - | - | - |
| ALT | rs13102451 (proxy of rs6834314) | 4chr | *-* | - | - | - |
| ALT | rs738409 | 22q13 | *PNPLA3, SAMM50* | Nonalcoholic fatty liver disease(NFLD), Cirrhosis (alcohol related) | Cirrhosis (alcohol related), Nonalcoholic fatty liver disease | + |
| ALP | rs10819937 | 9q21 | *ALDOB, C9orf125* | - | - | - |
| ALP | rs16856332 | 2q24 | *ABCB11* | - | - | - |
| ALP | rs174601 | 11q12 | *C11orf10, FADS1, FADS2* | Blood metabolite levels, TC, Gondoic acid (20:1n-9) levels, HDL, Red blood cell fatty acid levels, Trans fatty acid levels | Gondoic acid levels, Trans fatty acid levels, Red blood cell fatty acid levels, Blood metabolite levels | - |
| ALP | rs1883415 | 6p22 | *ALDH5A1, GPLD1* | - | - | - |
| ALP | rs1976403 | 1p36.12 | *ALPL, NBPF3* | - | - | - |
| ALP | rs2236653 | 11q.24 | *ST3GAL4* | - | - | - |
| ALP | rs281377 | 19q13 | *FUT2* | Resting metabolic rate | Yeast infection, Resting metabolic rate | + |
| ALP | rs2954021 | 8q24 | *TRIB1* | TC, HDL, LDL, ALT, Lymphocyte percentage of white cells, Neutrophil percentage of white cells, Response to fenofibrate (triglyceride levels) | Triglyceride levels, ALT, LDL | + |
| ALP | rs314253 | 17p13 | *ASGR1, DLG4* | TC, LDL | LDL cholesterol levels, Total cholesterol | - |
| ALP | rs579459 | 9q34 | *ABO* | Blood metabolite ratios, C-reactive protein levels , TC, Coronary Artery Disease, Ischemic stroke, Large artery stroke, E-Selectin, LDL, Red blood cell count, Red blood cell traits, Soluble E-selectin levels, Soluble levels of adhesion molecules, Urinary metabolites (H-NMR features), | Glycated hemoglobin levels, Total cholesterol, LDL, Soluble levels of adhesion molecules, Red blood cell count, Urinary metabolites (H-NMR features), Coronary artery disease, Coronary artery disease or large artery stroke, Coronary artery disease or ischemic stroke, Coronary heart disease, Red blood cell traits, Blood metabolite ratios | + |
| ALP | rs6984305 | 8p23 | *PPP1R3B* | TC, HDL | - | - |
| ALP | rs7186908 | 16q22 | *HPR, PMFBP1* | - | - | - |
| ALP | rs7267979 | 20p11 | *ABHD12,GINS1, PYGB* | - | - | - |
| ALP | rs7923609 | 10q21 | *JMJD1C, NRBF2* | Educational attainment | Educational attainment | - |
| GGT | rs10513686 | 3q26 | *SLC2A2* | - | - | - |
| GGT | rs1076540 | 22q11.21 | *MICAL3* | - | - | - |
| GGT | rs10908458 | 1q21 | *DPM3, EFNA1, PKLR* | - | - | - |
| GGT | rs12145922 | 1p22 | *CCBL2, PKN2* | - | - | - |
| GGT | rs1260326 | 2p23 | *C2orf16, GCKR* | Blood metabolite levels, C-reactive protein levels, Triglyceride levels, Caffeine metabolism (plasma 1,7-dimethylxanthine (paraxanthine) to 1,3,7-trimethylxanthine (caffeine) ratio), Cardiovascular disease risk factors, TC, Chronic kidney disease, Coffee consumption , Crohn's disease , Fasting Glucose (More seen in http://www.ensembl.org) | Alcohol consumption, Triglyceride, Crohn's disease, Inflammatory bowel disease, Plasma lactate levels, Hypertriglyceridemia,Renal overload goutBlood metabolite levels, Gout, Non-albumin protein levels, Two-hour glucose challenge (More could be assessed in https://www.ebi.ac.uk/gwas/search?query=rs1260326 ) | + |
| GGT | rs12968116 | 2q37 | *ATP8B1* | Body Height, Familial Intrahepatic Cholestasis | - | - |
| GGT | rs13030978 | 2q12 | *MYO1B, STAT4* | - | - | - |
| GGT | rs1335645 | 1p13 | *CEPT1* | - | - | - |
| GGT | rs1497406 | 1p36.13 | *RSG1, EPHA2* | - | - | - |
| GGT | rs17145750 | 7q11 | *MLXIPL* | Metabolite levels (lipoprotein measures), Platelet Count | Platelet count, Metabolite levels (lipoprotein measures) | - |
| GGT | rs2073398 | 22q11.23 | *GGT1, GGTLC2* | - | - | - |
| GGT | rs5751901 (proxy of rs2073398) | 22q11.23 | *GGT1* | Protein quantitative trait loci | GGT, Hematological and biochemical traits, Cardiovascular disease risk factors |  |
| GGT | rs2140773 | 2q37 | *EFHD1, LOC100129166* | - | - | - |
| GGT | rs2739330 | 22q11.23 | *DDT, DDTL, GSTT1, GSTT2B, MIF* | - | - | - |
| GGT | rs339969 | 15q21 | *RORA* | - | - | - |
| GGT | rs4074793 | 5q11 | *ITGA1* | - | - | - |
| GGT | rs4503880 | 18q21.32 | *NEDD4L* | - | - | - |
| GGT | rs4547811 | 4q31 | *ZNF827* | - | - | - |
| GGT | rs4581712 | 16q23 | *DYNLRB2* | - | - | - |
| GGT | rs516246 | 16q23 | *FUT2* | TC, Crohn's disease (time to surgery), Inflammatory bowel disease, Obesity-related traits | Crohn's disease, Inflammatory bowel disease, Obesity-related traits | + |
| GGT | rs6888304 | 5p15 | *CDH6* | - | - | - |
| GGT | rs7310409 | 12q24 | *HNF1A, C12orf27* | C-reactive protein, Pancreatic Cancer, Pancreatic Neoplasms | Pancreatic cancer, C-reactive protein | - |
| GGT | rs754466 | 10q23 | *DLG5* | - | - | - |
| GGT | rs8038465 | 15q23 | *CD276* | - | - | - |
| GGT | rs9296736 | 6p12 | *MLIP* | - | - | - |
| GGT | rs944002 | 14q32 | *EXOC3L4* | Mean platelet volume | Mean platelet volume | - |
| GGT | rs2297067 (proxy of rs944002) | 14chr | *-* | Platelet Count, Primary biliary cholangitis | Primary biliary cholangitis, Platelet count | - |
| GGT | rs9913711 | 17q24 | *FLJ37644, SOX9* | - | - | - |

ALT: alanine aminotransferase; ALP: alkaline phosphatase; GGT: gamma glutamyltransferase

TC: total cholesterol; HDL: high density lipoprotein cholesterol; LDL: low density lipoprotein cholesterol

Supplemental Table S3. Characteristics of palindromic single nucleotide polymorphisms (SNPs) in the exposure and outcome genome-wide association studies (GWAS).

| Phenotype | SNP | Effect Allele | Other Allele | EAF | EAF_GIANT | EAF_GIANTUKB |
| --- | --- | --- | --- | --- | --- | --- |
| ALT | rs10883437 | T | A | 0.64 | 0.62 | 0.61 |
| ALT | rs738409 | G | C | 0.23 | 0.23 | 0.23 |
| ALP | rs10819937 | C | G | 0.17 | 0.14 | 0.19 |
| ALP | rs6984305 | A | T | 0.11 | 0.11 | 0.11 |
| ALP | rs7186908 | C | G | 0.24 | 0.18 | 0.2 |
| GGT | rs2073398 | G | C | 0.34 | 0.39 | NA |
| GGT | rs754466 | T | A | 0.24 | 0.29 | 0.26 |
| GGT | rs9913711 | C | G | 0.65 | 0.68 | 0.66 |

ALT: alanine aminotransferase; ALP: alkaline phosphatase; GGT: gamma glutamyltransferase

EAF: effect allele frequency obtained from Chambers et al., 2011

EAF_GIANT: effect allele frequency obtained from GWAS Anthropometric 2015 BMI and GWAS Anthropometric 2015 Waist

EAF_ GIANTUKB: effect allele frequency obtained from the 2018 GIANT and UK Biobank meta-analysis

NA: no available data in specific dataset

Supplemental Table S4. Characteristics of unequivocally aligned single nucleotide polymorphisms (SNPs) in the exposure and outcome genome-wide association study (GWAS).

| Phenotype | SNP | EA | OA | EAF_E | Proxy | Source of proxy | R^2^¶ | EA_P | OA_P | EAF_P¶ | EA_O | OA_O | EAF_O | Outcome dataset | Beta in outcome | Standard error in outcome |
| --- | --- | --- | --- | --- | --- | --- | --- | --- | --- | --- | --- | --- | --- | --- | --- | --- |
| ALT | rs6834314 | A | G | 0.75 | - | - | - | - | - | - | NA | NA | NA | WC-Female | NA | NA |
|  |  |  |  |  | rs13102451 | Exposure GWAS* | 1.00/1.00 | A | G | 0.75/- | A | G | 0.72 | WC-Female | -0.0083 | 0.0061 |
| GGT | rs944002 | G | A | 0.21 | - | - | - | - | - | - | NA | NA | NA | WC-Male/WHR-Male | NA | NA |
|  |  |  |  |  | rs2297067 | Exposure GWAS* | 0.98/1.00 | T | C | 0.24/0.19 | T | C | 0.2 | WC-Male | -0.0008 | 0.0062 |
|  |  |  |  |  |  |  |  |  |  |  |  |  |  | WHR-Male | 0.0062 | 0.0063 |
| GGT | rs2073398 | G | C | 0.34 | - | - | - | - | - | - | NA | NA | NA | GIANTUKB | NA | NA |
|  |  |  |  |  | rs5751901 | Exposure GWAS* | 0.95/0.96 | C | T | -/0.37 | C | T | 0.35 | GIANTUKB | 0.0046 | 0.0021 |
| GGT | rs516246 | T | C | 0.47 | - | - | - | - | - | - | NA | NA | NA | GIANTUKB | NA | NA |
|  |  |  |  |  |  |  |  |  |  |  | T | C | 0.54 | GIANT | Ф | Ф |
| GGT | rs8038465 | T | C | 0.39 | - | - | - | - | - | - | T | C | 0.41 | GIANTUKB | 0.0029 | 0.0020 |
|  |  |  |  |  |  |  |  |  |  |  |  |  | 0.57 | GIANT | Ф | Ф |

* Proxy SNPs were from the GWAS of Chambers et al., 2011

¶ The front estimate was obtained from LDlink and the following estimate was the GWAS from Chambers et al., 2011

Ф Values could not be given since the outcome has not been specified

WC-Male, WC-Female and WHR-Male are of the GWAS Anthropometric 2015 Waist

GIANT is of GWAS Anthropometric 2015 BMI and the GWAS Anthropometric 2015 Waist

GIANTUKB is of the 2018 GIANT and UK Biobank meta-analysis

NA: no available data in specific dataset;

WC: waist circumference, WHR: waist-hip ratio

EA: effect allele of the original SNP; OA: other allele of the original SNP; EAF_E: effect allele frequency of the original SNP in the exposure GWAS;

EA_P: effect allele of the proxy SNP; OA_P: other allele of the proxy SNP; EAF_P: effect allele frequency of the proxy SNP;

EA_O: effect allele of the proxy SNP in the outcome GWAS; OA_O: other allele of the proxy SNP in the outcome GWAS; EAF_O: effect allele frequency of the proxy SNP in the outcome GWAS
